# A group-based mental health intervention for young people living with HIV in Tanzania: results of a pilot individually randomized group treatment trial

**DOI:** 10.1186/s12889-020-09380-3

**Published:** 2020-09-04

**Authors:** Dorothy E. Dow, Blandina T. Mmbaga, John A. Gallis, Elizabeth L. Turner, Monica Gandhi, Coleen K. Cunningham, Karen E. O’Donnell

**Affiliations:** 1 Duke University Medical Center, Pediatrics, Infectious Diseases, Box 3499, Durham, NC 27710 USA; 2 Duke Global Health Institute, Durham, NC USA; 3 Kilimanjaro Christian Medical Centre, Moshi, Tanzania; 4 Duke University, Department of Biostatistics and Bioinformatics, Durham, NC USA; 5 University of California, San Francisco, USA; 6 Duke University, Center for Health Policy and Inequalities Research, Durham, NC USA; 7 Center for Child and Family Health, Durham, NC USA

**Keywords:** Adolescent, Young people, HIV, Mental health, Africa, Intervention, ART adherence, HIV RNA, Viral load, Tanzania

## Abstract

**Background:**

Increasing numbers of young people living with HIV (YPLWH) have unaddressed mental health challenges. Such challenges are associated with poor antiretroviral therapy (ART) adherence and high mortality. Few evidence-based mental health interventions exist to improve HIV outcomes among YPLWH.

**Methods:**

This pilot group treatment trial individually randomized YPLWH from two clinical sites in Tanzania, evaluated acceptability, feasibility, and preliminary effectiveness of a mental health intervention, *Sauti ya Vijana (SYV*; The Voice of Youth*),* was compared to the local standard-of-care (SOC) for improving ART adherence and virologic suppression. Enrolled YPLWH were 12–24 years of age and responded to mental health and stigma questionnaires, self-reported adherence, objective adherence measures (ART concentration in hair), and HIV RNA at baseline and 6-months (post-intervention). Feasibility and acceptability were evaluated, and potential effectiveness was assessed by comparing outcomes between arms using mixed effects modeling.

**Results:**

Between June 2016 and July 2017, 128 YPLWH enrolled; 105 were randomized and 93 (55 in SYV) followed-up at 6-months and were thereby included in this analysis*.* Mean age was 18.1 years; 51% were female; and 84% were HIV-infected perinatally. Attendance to intervention sessions was 86%; 6-month follow-up was 88%, and fidelity to the protocol approached 100%. Exploratory analyses of effectiveness demonstrated self-reported adherence improved by 7.3 percentage points (95% CI: 2.2, 12.3); and the pooled standard deviation for all ART concentration values increased by 0.17 units (95% CI: − 0.52, 0.85) in the *SYV* arm compared to SOC. Virologic suppression rates (HIV RNA < 400 copies/mL) at baseline were 65% in both arms but increased to 75% in the *SYV* arm while staying the same in the SOC arm (RR 1.13; 95% CI: 0.94, 1.36).

**Conclusions:**

YPLWH often have poor HIV outcomes, making interventions to improve outcomes in this population critical. This pilot trial of the Tanzania-based *SYV* intervention demonstrated trends towards improvement in ART adherence and virologic outcomes among YPLWH, supporting efforts to scale the intervention into a fully-powered effectiveness trial.

**Trial registration:**

ClinicalTrials.gov Identifier: NCT02888288. Registered August 9, 2016. Retrospectively registered as first participant enrolled June 16, 2016.

## Background

Scale-up of antiretroviral therapy (ART) has enabled growing numbers of HIV-infected children to survive to adolescence and beyond. Despite increase in ART access, a young person (age 10–24 years) dies every 10 min due to an AIDS related illness [1]. Adolescence brings unique challenges with hormone surges, changing bodies, desire for independence, less inhibition, and prioritization of peer networks and “fitting in” over adhering to medication and health [2]. Young people living with HIV (YPLWH) have the added burden of navigating peer and romantic relationships while living with a stigmatizing and sexually transmittable disease that requires adherence to daily treatment. These factors contribute to worse HIV outcomes among youth when compared to children or adults [3, 4]. The increasing number of youth living with HIV worldwide, known as the youth bulge, and their poor virologic outcomes endanger the progress towards eliminating AIDS as a public health threat by 2030 [5, 6].

Reasons for high mortality among YPLWH are largely due to delays in diagnosis [7] and inadequate ART adherence [8]. Contributors to inadequate adherence in young people include mental health difficulties [9–11]. Half of all mental health conditions have been reported to start by the age of 14, most which go undetected and untreated [12]. Across two sites in Moshi, Tanzania, 25–45% of YPLWH self-reported significant difficulties with depression, post-traumatic stress, and/or emotional/behavioral challenges [9, 13]. Prior studies have demonstrated that mental health interventions can effectively improve ART adherence in high income countries [14, 15] and proof of concept has been demonstrated for adults in low resource settings [16–18]. However, evidence-based interventions designed to address the unique mental health challenges faced by YPLWH in low resource settings are lacking [19, 20].

We developed *Sauti ya Vijana (SYV; The Voice of Youth)*, a group-based, scalable, mental health intervention delivered by young adult group leaders to address the mental health challenges previously reported by YPLWH in Tanzania [9, 21]. SYV was conceptualized to be broadly inclusive to help prevent and/or reduce the severity of social-emotional distress by cultivating coping strategies and family support to bolster resilience in YPLWH and help them view ART adherence as a critical strategy for living positively with HIV [22]. Our hypothesized mechanism of change was that SYV would reduce mental health difficulties and internal stigma and that these potential mediators would lead to improved ART adherence and therefore virologic suppression [9].

The primary objective of this pilot trial was to establish the feasibility and acceptability of the *SYV* intervention in preparation for a fully powered effectiveness trial [22]. The secondary objective was to conduct exploratory analyses of the impact of SYV on other outcomes, including mental health, stigma, ART adherence, and HIV RNA.

## Methods

### Study design and participants

The study was an individually-randomized group treatment pilot trial that used a stepped-wedge design across three waves of young people (Supplemental Figure 1). The current report includes data on the outcomes at baseline and 6-months for each wave. One male and one female group from each site are included per wave and groups were filled on a rolling basis through July 2017. Our plan was to have groups including ages 12–14/15 years; 15–19 years; 18/19–24 years, but not enough youth under 15 years enrolled to create the younger groups. The mean age and age range for each group can be seen in Supplemental Figure 1.

YPLWH 12–24 years of age who attended the established adolescent HIV clinics at Kilimanjaro Christian Medical Centre (KCMC) or Mawenzi Regional Referral Hospital (MRRH), both located in Moshi, Tanzania, were approached to participate. Full HIV disclosure was required to attend either clinic, thus all patients attending clinic were fully aware of their HIV status. This was confirmed by each participant as part of the consent process. Inclusion criteria included the ability to understand and participate in the study intervention and receipt of ART. ART is prescribed and obtained by the clinic pharmacy free of charge during adolescent HIV clinic. Young people with cognitive disabilities precluding active participation in the consent process, intervention, or study assessments were excluded. There were no changes to the eligibility criteria or methods during the pilot trial.

Participants 18 years or older provided written informed consent for themselves; those under 18 years provided written assent with written consent provided by their caregivers. Caregivers were not treated as participants in the study and did not provide study data. Ethical approval for the study was granted from Duke University, KCMC, and the National Institute of Medical Review in Tanzania.

Study methods and results reporting followed the 2010 CONSORT checklist for reporting a pilot or feasibility trial [23]. Clinicaltrials.gov outlines the protocol (NCT 02888288) and a full study protocol is available upon request from the corresponding author.

### Procedures

To provide sufficient information on the feasibility and acceptability of implementing the intervention and perform preliminary estimates of intervention effects, the targeted enrollment was 108–120 participants in 6 groups of 18–20 young people randomized per group and two groups per wave (see supplemental Figure 1). Due to attrition, enrollment was increased to 130 to meet the target sample size.

From May to July 2016, YPLWH were recruited via a study announcement made by study staff during monthly adolescent HIV clinic at KCMC and MRRH. Interested young people were invited to join a study waitlist that included their name, age, sex, site, and contact information. Young people were then grouped by age, sex, and clinic site in the order in which they signed the waitlist, and the first 60 from each site that made up groups of 18–20 with similar age and same sex were contacted to confirm their commitment to attend all intervention sessions and study visits. Young people were encouraged to invite their caregiver to attend two joint caregiver sessions, but enrollment was not contingent on caregiver participation. Young people who were available to participate and to provide informed written consent/assent were enrolled. Enrollment was not limited to only YPLWH who endorsed mental health challenges on baseline screening in order to establish acceptability and feasibility across a wide spectrum of mental health challenges and the hypothesis that all YPLWH could benefit from the intervention.

At enrollment and subsequent study visits (Supplemental Figure 1), participants responded to a structured interview conducted by one of two research assistants in Kiswahili. The interview included questions about demographics, perception of stigma, ART adherence, risk behaviors, and mental health symptoms. A hair sample was obtained to measure ART drug concentration as a cumulative measure of ART adherence and a five mL blood sample was drawn to measure HIV RNA. The participant file was reviewed to document the date the participant began ART, current ART regimen, most recent CD4 cell count, and (when available) the most recent standard-of-care HIV RNA result.

### Randomization and masking

Once a male and female group of 18–20 young people of similar age, same sex, and same site were enrolled, they formed a “wave”. Each wave of individuals in the male and female groups were randomized by coin flip in the presence of the principal investigator and group leaders. Participants randomized to the *SYV* arm received the intervention; participants randomized to SOC continued with usual attendance to the monthly adolescent HIV clinic, but the SOC arm did not meet together as a study group. SOC, as provided to both study arms, included routine adherence counseling that was uniform across both sites and followed national Tanzanian guidelines [24]. All youth received adherence counseling in the form of health talks provided during the routine clinic; and, per guidelines, those with virologic failure (HIV RNA > 1000 copies) met with an adherence nurse on a monthly basis for three months to document youth adherence, discuss barriers to adherence, and provide support and suggestions to improve adherence. No mental health screening nor mental health specialists were available for referral, thus no specific mental health referrals were made at either clinic. The two research assistants conducting the study visits were blinded to the participant’s study arm assignment.

### Intervention

SYV was developed based on our formative research that identified the common mental health needs of YPLWH in Tanzania [9, 21]. We demonstrated that 32% of YPLWH reported symptoms of mental health difficulties (depression, post-traumatic stress, and emotional/behavioral symptoms) as measured quantitatively with the same measures utilized in this study and described in detail below [9, 13, 22]. Components of the SYV intervention were adapted, in part, from a study of a cognitive behavioral therapy for bereaved orphans in Tanzania that reported improved outcomes on indicators of grief, post-traumatic stress, depression, and overall behavioral adjustment that were sustained at 3 and 12 months [25–27]. SYV was tailored further to the needs of YPLWH with additional components of interpersonal psychotherapy [28] and motivational interviewing [29] to address the specific challenges identified by those YPLWH in the formative study [9, 21]. The idea was to be responsive to the needs identified by this group of youth while preserving aspects of existing evidence-based treatment models. The group intervention involved ten group sessions, two designed to be held jointly with caregivers, and two individual sessions between the participant and one group leader (Fig. 2). A trauma narrative: “How I learned I have HIV” was conducted first in an individual session with the group leader to discuss their memories and then to prepare for what, if any, aspects of the disclosure event the participant wanted to share with other group members and/or caregivers. Caregivers were invited to participate in the first *SYV* session to understand the intervention content and share common challenges with other caregivers of YPLWH. In the sixth session, caregivers were asked to attend to hear the young person’s narrative about learning about HIV-infection from the young person’s perspective. A description of the scripted session-by-session intervention protocol has been previously described [22], and the full intervention is available upon request to the corresponding author or at sites https://duke.edu/sautiyavijana.

The study was implemented with Tanzanian group leaders trained to deliver the intervention and who were closely supervised twice weekly. Six young adults (age 23 to 30 years) with a background of either living with HIV and/or having prior experience with mental health research were provided an on-site intensive two-week training with the principal investigator and the U.S.-based clinical psychologist, both of whom provided subsequent weekly supervision for each session. Sessions lasted approximately 90 min and were held on Saturdays at a centrally-located site not affiliated with HIV care, providing a non-clinical space where young people could be comfortable discussing topics freely. Enrolled young people who missed more than two intervention sessions were not allowed to continue as content and group rapport builds from the previous sessions. These young people were offered the option to join a future wave as part of a cross-over group in the stepped-wedge design (Supplemental Figure 1).

### Measures

All measures were administered in Kiswahili, the official language of Tanzania. Two different professional translators fluent in both Tanzanian Kiswahili and US English were involved. One translated the original English to Kiswahili, and the other back translated the Kiswahili to English. The back translated and original English were compared and clarifications incorporated. The formative study acted as the pilot of the mental health and stigma measurements [9].

Acceptability and feasibility have been previously reported elsewhere, [22] but did not include 6-month follow -up data as presented here. Measures of acceptability and feasibility included recruitment, attendance and participation during *SYV* sessions, follow up visits, and the ability of the group leaders to maintain fidelity to the intervention content based on supervisor observation and as documented on fidelity checklists for each session. Adherence to the scripted SYV manual was documented by a group leader observing (but not leading) each session. Fidelity checklists were based on intervention content whereby the observing group leader marked if the content was covered and documented the amount of time utilized in discussion. If content was skipped, the reason was documented and the content was revisited at a later session. The fidelity checklist was designed by the intervention developers (O’Donnell and Dow, Supplemental Table 2). Session notes were also documented by the observer noting each participant’s demeanor and participation in the group discussion. Fidelity checklists (quantitative data) and session notes (qualitative data) were discussed during the weekly post-session debrief meetings between the supervisors (Dow, Mmbaga, and O’Donnell) and group leaders and also ad hoc (as needed).

The *Patient Health Questionnaire* (PHQ-9) was used to measure symptoms of depression. The PHQ-9 has been validated relative to other diagnostic tools in several studies in Africa, [30, 31] including adolescent populations in Kenya and Ghana [32, 33] demonstrating high internal consistency with Chronbach alphas of > 0.70 [32, 34]. This measure includes nine questions with a response range of 0–27 with a score of 10 or greater used as a screening threshold suggestive of moderate to severe depressive symptoms [30, 31] and was used in our prior research [9]. The *Strengths and Difficulties Questionnaire* (SDQ) was used to measure young people’s emotional and behavioral symptoms. The scale includes 25 questions (5 prosocial behavior questions are not included in the composite score). A cutoff of 17 or higher (score range of 0–40) signals symptomatology of mental health difficulties with mean Cronbach α: 0.73 as assessed by the tool developers [35]. Trauma related symptoms were assessed using the *UCLA Post Traumatic Stress Symptoms Exposure Screener and Reaction Index* survey [36, 37] modified to a four-question Likert scale (None, Some, Much, and Most) based on Swahili translation as previously described with a cutoff of 18 or greater [9]. Prior use of the tool internationally demonstrated inter-rater reliability and criterion-related validity with children in Zambia [38] and demonstrated internal consistency among adolescent Somalian refugees (Cronbach α = 0.85) [39].

Stigma was measured using a truncated (10 question) *Berger HIV Stigma Scale*. Included questions related to both internal or ‘negative self-image’ (four questions, 2,3,7,12) and external stigma or ‘public attitudes’ (six questions, 9, 10, 14, 16, 20, 32) and were analyzed in these two categories as well as the total score for interpreting results (scale range 10–40) [40] and were used in our prior research [9].

Oral interviews were documented by group leader notes as part of the two individual sessions. Similarly, group discussion notes were documented by the group leader observer during intervention sessions.

ART adherence was measured by a validated three-question survey that asked participants to rate their adherence in the past 30 days transformed to a 0–100 scale [41]. The mean of these three items was the value used to measure self-reported adherence with a higher number indicating better adherence. A fourth open-ended question asked, “Many people have trouble remembering to take their medication. Please think about the last time you missed your medication. What was the reason?” in order better understand adherence challenges from the participant’s perspective. An objective metric of adherence was assessed by analyzing ART concentrations in hair. Hair samples (20–30 strands) were collected and concentrations of efavirenz (EFV), nevirapine (NVP), lopinavir (LPV), atazanavir (ATV) and ritonavir (RTV) were measured at the University of California, San Francisco (UCSF) Hair Analytical Laboratory (HAL) using validated, reproducible, liquid chromatography–tandem mass spectrometry (LC-MS/MS)-based methods [42]. For levels below the limit of quantitation (BLQ), a value of 0.05 (the BLQ equivalent) was used [43, 44]. Only participants contributing hair samples and receiving the same ART regimen at both baseline and the six-month time points are included in the analysis of hair data.

HIV RNA measurement was performed at the Kilimanjaro Clinical Research Institute Biotechnology Laboratory, which participates in international external quality assurance programs, using the Abbott m2000 platform (Des Plaines, Illinois, USA). Virologic failure was defined as plasma HIV RNA level > 400 copies/ml. HIV RNA lower than the minimal detectable amount of 40 copies/ml were imputed as half the minimum detectable amount (20 copies/ml).

### Statistical analysis

Continuous variables were summarized using means and standard deviations (SD), and categorical variables were summarized using counts and percentages. Data were analyzed as intention-to-treat according to the participant’s randomization assignment, regardless of whether the participant attended or participated in the intervention if assigned to the intervention arm. Item nonresponse resulting in missing data was < 3%. If a question was skipped within a quantitative measure (ex. PHQ-9 or Stigma) the entire measure was treated as missing. Analyses were also run using imputed data of missing items with the mean of answered items and it did not appreciably change any of the results.

In the exploratory analyses for each measure, we fitted mixed effects models with a random intercept for group in the treatment arm to take into account the group treatment effects. Residual errors were allowed to vary by arm [45]. For each continuous outcome, we modeled the change in the outcome from baseline to six-month follow-up, adjusting for the baseline level of the outcome, wave, and site as covariates. To create a standardized hair measure collapsing the four anchor antiretrovirals (EFV, NVP, LPV, ATV) into a single measure, we defined a variable that was the log base 2 of the hair level minus the median log base 2 of the hair level for each drug and combined these “standardized” hair values into a single variable [43]. All analyses were conducted using Stata version 15.1 (StataCorp, College Station, TX).

## Results

**Fig. 1:**
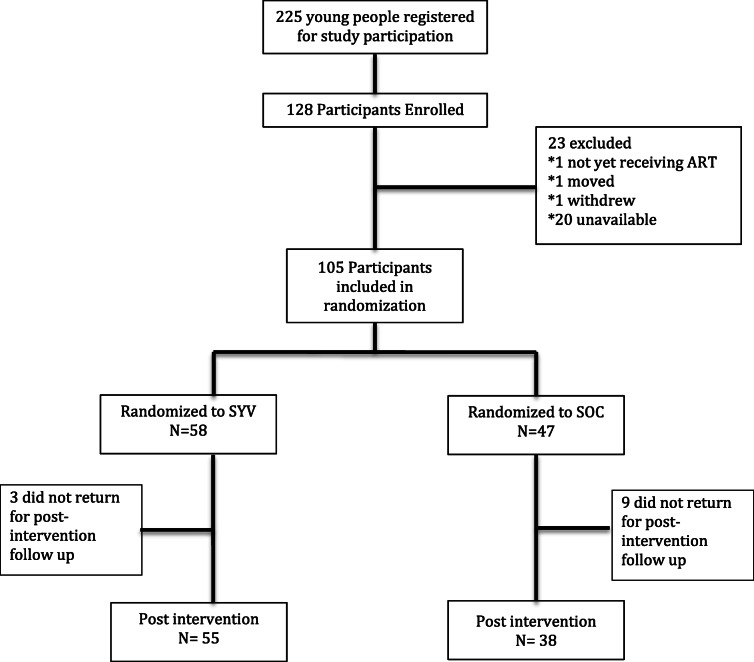
CONSORT flow diagram

Of the 225 young people who signed the study waitlist during recruitment, 128 were enrolled. Some were no longer able to commit to intervention attendance in wave two and three, so only 105 (82%) were randomized. Of those randomized, 93 (55 in the SYV arm and 38 in the SOC arm) attended both the baseline and six-month study visits and were included in this analysis (Fig. 1). Reasons participants missed the study visit included those not reachable, busy with school exams, or moved. There were no significant differences in characteristics between the 23 participants who were enrolled but were not randomized compared to the 105 enrolled and randomized or to the 12 who did not provide six-month data (Supplemental Table 1). Of the 93 participants with both baseline and six-month data, approximately half (51%) were female and mean age was 18.1 years. The majority were confirmed to be HIV-infected perinatally (84%), and 78% reported that one or both parents had died. One-half of young people lived with a biologic parent as their caregiver, and 25% were neither in school nor working (Table 1).

**Table 1:** Baseline characteristics of participants with both baseline and follow-up measurements (*N* = 93)^a^

		Standard of care *N* = 38	SYV intervention *N* = 55	Total N = 93
Gender (female)		21 (55%)	26 (47%)	47 (51%)
Age^b^		18.5 (2.2)	17.7 (2.2)	18.1 (2.3)
Primary Caregiver	Biological parent	18 (47%)	29 (53%)	47 (51%)
	Grandparent	2 (5%)	12 (22%)	14 (15%)
	Aunt/Uncle	12 (32%)	11 (20%)	23 (25%)
	Sibling	3 (8%)	2 (4%)	5 (5%)
	Other	3 (8%)	1 (2%)	4 (4%)
Orphan status	Both parents alive	8 (21%)	15 (27%)	23 (25%)
	Single orphan	22 (58%)	28 (51%)	50 (56%)
	Double orphan	8 (21%)	12 (22%)	20 (22%)
Perinatal HIV Transmission		31 (82%)	47 (86%)	78 (84%)
Antiretroviral therapy (ART)	Years receiving ART^b^	7.72 (3.65)	7.33 (3.27)	7.49 (3.41)
	Nevirapine (NVP)	10 (26.3%)	9 (16.4%)	19 (20.4%)
	Efavirenz (EFV)	14 (36.8%)	23 (41.8%)	37 (39.8%)
	Lopinavir/ritonavir (LPV/r)	7 (18.4%)	10 (18.2%)	17 (18.3%)
	Atazanavir/ritonavir (ATV/r)	7 (18.4%)	13 (23.6%)	20 (21.5%)
Livelihood^c^	In School	28 (74%)	37 (67%)	65 (70%)
	Working	5 (14%)	6 (11%)	11 (12%)
	Both	1 (3%)	2 (4%)	3 (3%)
	Neither	8 (21%)	15 (27%)	23 (25%)
Home Environment^c^	Electricity	30 (79%)	39 (71%)	69 (74%)
	Indoor plumbing	22 (60%)	39 (71%)	61 (66%)
	Both	21 (55%)	33 (60%)	54 (58%)
	Neither	8 (21%)	10 (18%)	18 (19%)
Own a Cell Phone		27 (71%)	35 (64%)	62 (67%)
Behaviors	Report being sexually active	14 (37%)	13 (24%)	27 (29%)
	Age at sexual debut^b^	16.9 (4.2)	15.7 (1.7)	16.4 (3.3)
	Reports condom use with latest sexual encounter^d^	9 (64%)	10 (77%)	19 (70%)
	Report consuming alcohol/other drugs	4 (11%)	4 (7%)	8 (9%)

^a^Counts (percentages) are reported unless otherwise noted; baseline characteristics of the full study sample (*n* = 105) can be found in Supplementary Table 1

^b^Mean (standard deviation)

^c^Those listed in the “both” category are also enumerated in the individual categories

^d^Denominator is those who report sexual activity

### Acceptability and feasibility

Attendance at the six-month follow-up visit was 89% (93/105) with 95% among those in the intervention arm and 81% among those receiving SOC. Four participants randomized to receive the intervention never attended a session, two of whom came for the six-month follow-up visit. Three participants missed more than two sessions and therefore were unable to complete the intervention program, but all participated in the six-month follow-up. Overall attendance to intervention sessions, including those who never attended or dropped after missing more than two sessions, was 86%. Of the 13 possible sessions, 10 group, two individual, and final celebration, participants attended an average of 11 sessions including data from those who never came or were dropped. During the final individual session, the vast majority of participants described how much the group participation improved their outlook on life and acceptance of their situation living with HIV. They reported that they planned to teach their friends and community about what they learned. This was witnessed by members of the youth community advisory board and health care workers during adolescent HIV clinic. Fidelity to the intervention was maintained as evidenced by fidelity checklists (for an example see supplemental Table 2) and session notes demonstrating that group leaders followed the script and elicited rich discussion from participants. No content in the fidelity checklists was skipped. No harms were identified as part of the study.

### Exploratory effectiveness outcomes

At baseline, 10 (27%) of participants in the SOC arm compared to 4 (7%) in the intervention arm met criteria for moderate symptoms of depression based on the PHQ-9 cutoff of 10 or greater; however, the mean score for PHQ-9 and all other mental health measures was below the screening cutoff in both arms (Table 2). After adjusting for baseline, sex, and site, mental health scores were reduced more in the intervention arm compared to the SOC arm for all mental health measures (Table 3). High stigma scores were common in both arms, and data from both arms reported decreased internal stigma at six-months, though the change was minimal (0.2 in the intervention arm). During the final individual meeting, however, more than half of participants described how prior to the intervention they were unaware of internal stigma and now they accept their situation of living with HIV. External stigma was reduced at six-months for the SOC arm, but increased in the intervention arm. The combined stigma score demonstrated a 2.11 (95% CI = 0.25, 3.98) unit increase in stigma in the intervention arm compared to SOC (Table 3).

**Table 2:** Mental health and HIV measures at baseline and 6-month follow-up, for participants with data at both time points (N = 93)^a^

		Standard of care (N = 38)			*SYV* Intervention (N = 55)		
		Baseline	6-month	Change	Baseline	6-month	Change
PHQ-9^f^	Total score	6.3 (4.1)	5.1 (3.9)	− 0.9 (3.7)	4.9 (3.3)	4.1 (3.4)	− 0.8 (4.0)
	≥ 10^b^	10 (27.0%)	4 (11.1%)		4 (7.3%)	5 (9.1%)	
SDQ^f^	Total score	7.1 (3.6)	7.3 (4.4)	0.1 (3.9)	7.3 (4.0)	6.7 (4.4)	− 0.6 (3.9)
	≥ 17^b^	0 (0.0%)	2 (5.6%)		0 (0.0%)	2 (3.6%)	
UCLA Trauma^f^	Total score	9.3 (6.9)	8.9 (6.3)	−0.3 (5.9)	8.6 (7.4)	8.6 (7.5)	0.0 (7.3)
	≥ 18^b^	5 (13.5%)	4 (10.8%)		9 (16.4%)	8 (14.5%)	
Stigma^f^ Mean (SD)	Total Score	23.5 (3.7)	21.5 (5.1)	−2.3 (4.7)	21.9 (5.1)	22.7 (5.3)	0.6 (4.2)
	Internal Score	8.1 (1.8)	7.1 (2.0)	−1.1 (1.7)	7.7 (1.9)	7.5 (2.1)	−0.2 (2.5)
	External Score	15.5 (3.7)	14.3 (4.2)	−1.2 (3.9)	14.4 (4.3)	15.1 (4.5)	0.7 (3.3)
Adherence^f^	Self-report Score^d^	57.7 (15.2)	57.7 (14.9)	−0.1 (12.4)	60.5 (11.6)	65.5 (12.3)	5.1 (17.0)^f^
ART concen-tration in Hair (ng/mg)	NVP (*N* = 13)	44.2 (15.4)	49.7 (20.8)	5.4 (16.1)	47.1 (19.2)	45.9 (20.0)	2.4 (32.2) ^f^
	EFV (*N* = 29)	9.3 (7.3)	10.0 (8.2)	−1.1 (4.1)	6.0 (5.4)	5.7 (3.3)	−0.3 (4.8)
	LPV (*N* = 15)	6.1 (5.9)	11.0 (11.7)	4.9 (9.2)	6.3 (6.6)	6.9 (5.9)	0.6 (6.5) ^f^
	ATV (*N* = 20)	4.7 (4.0)	4.4 (3.2)	−0.4 (2.3)	5.8 (3.8)	6.1 (3.1)	0.4 (1.7) ^f^
	Standardized (N = 77)^e^	−0.4 (2.0)	−0.5 (2.0)	− 0.1 (1.5)	−0.7 (2.3)	− 0.6 (1.7)	0.1 (2.1) ^f^
Viral Load Copies/mL	Total Score Log_10_	5.1 (2.8)	5.3 (3.3)	0.2 (1.8)	5.4 (3.3)	4.7 (2.6)	−0.7 (2.5)
	Virologic Suppression^b^ (<400 copies/mL)	25 (65.8%)	25 (65.8%)		35 (64.8%)	41 (74.5%)	

^a^Means (standard deviations) are reported unless otherwise noted; negative value favors the intervention except for adherence where a positive value signifies improved adherence

^b^Reported as count (percentage)

^d^Per (REF) has been scaled to a score of 0–100^31^

^e^ART concentration in hair available in 77/93; the lower limit of detection for EFV, LPV, ATV is 0.05 ng/mg and for NVP is 0.5 ng/mg

^f^Missing single item questionnaire response: Baseline, one participant for PHQ-9; 4 participants for SDQ; 1 participant for UCLA Trauma; 4 participants for stigma total score. At 6 months: 2 participants for PHQ-9; 1 participant for SDQ; 0 participants for UCLA Trauma; 1 participant for stigma total score. There is no item missingness at either time point for self-reported adherence

**Table 3:** Results of exploratory analyses estimating the difference between intervention and standard of care arms in change from baseline to 6 -months of key outcome variables^a^

	Adjusted for baseline value of outcome		Adjusted for baseline value of outcome, wave, and site	
Outcome	Estimated effect (95% CI)	ICC (95% CI)	Estimated effect (95% CI)	ICC (95% CI)
Change in PHQ-9 Score	−0.50 (− 2.08, 1.07)	0.067 (− 0.142, 0.276)	−0.60 (− 2.67, 1.47)	0.248 (− 0.142, 0.639)
Change in SDQ Total Difficulties Score	− 0.60 (− 2.18, 0.99)	< 0.001 (< 0.001, < 0.001)	−0.88 (−3.22, 1.47)	0.257 (− 0.168, 0.682)
Change in UCLA Trauma Score	0.03 (− 2.39, 2.45)	0.023 (− 0.145, 0.191)	−0.03 (− 2.38, 2.32)	0.025 (− 0.168, 0.217)
Change in Total Stigma Score	2.13 (0.12, 4.14)	0.069 (− 0.166, 0.304)	2.11 (0.25, 3.98)	< 0.001 (< 0.001, < 0.001)
Change in Internal Stigma Score	0.75 (− 0.13, 1.63)	0.084 (− 0.144, 0.311)	0.80 (− 0.12, 1.71)	0.089 (− 0.219, 0.397)
Change in External Stigma Score	1.53 (0.09, 2.97)	< 0.001 (< 0.001, < 0.001)	1.55 (0.09, 3.02)	< 0.001 (< 0.001, < 0.001)
Change in Self-Reported Adherence Score	6.73 (1.05, 12.41)	0.052 (− 0.126, 0.230)	7.29 (2.24, 12.33)	< 0.001 (< 0.001, < 0.001)
Change in Standardized hair concentration	0.12 (− 0.55, 0.79)	< 0.001 (< 0.001, < 0.001)	0.17 (− 0.52, 0.85)	< 0.001 (< 0.001, < 0.001)
Change in Log (Viral Load)	−0.81 (− 1.65, 0.02)	< 0.001 (< 0.001, < 0.001)	−0.84 (− 1.69, 0.01)	< 0.001 (< 0.001, < 0.001)

^a^ Negative value favors the intervention except for adherence where a positive value signifies improved adherence. Abbreviations: CI = Confidence Interval; ICC = Intracluster correlation

Being in the intervention arm was associated with a 7.29 (95% CI = 2.24, 12.33) unit increase in self-reported adherence compared to SOC (Table 3). The most common reasons participants reported missing ART included being in boarding school and fear of disclosure if seen taking medication, lack of transportation money to get to the clinic, being busy with exams or work, or simply forgetting. The biologic measure of adherence demonstrated increases in ART concentration for all drugs except EFV in the intervention group (Table 2 and Supplemental Figure 2). The overall change in the standard deviation of all hair ART concentration values was 0.17 units (95% CI = − 0.52, 0.85) greater in the intervention arm compared to SOC. Hair concentrations of ART were significantly higher in those who achieved virologic suppression compared to those with virologic failure (Table 4).

**Table 4:** Antiretroviral concentration in hair by viral suppression status at baseline and 6 -months (*N* = 77)^a^

	Baseline		6 -Months	
	Virologic suppression	Virologic failure	Virologic suppression	Virologic Failure
**Nevirapine (N = 13)**	48.37 (13.92) *N* = 11	19.65 (17.75) N = 2	51.46 (19.43) N = 11	30.35 (11.53) N = 2
**Efavirenz (*N*** **= 29)**	8.53 (6.50) *N* = 21^b^	2.53 (1.58) N = 7^b^	8.59 (6.16) *N* = 22	3.13 (2.46) N = 7
**Lopinavir (*N*** **= 15)**	9.56 (5.49) *N* = 8	2.57 (4.19) N = 7	13.85 (8.37) *N* = 9	1.30 (1.86) *N* = 6
**Atazanavir (*N*** **= 20)**	7.56 (2.96) N = 13	1.56 (1.69) N = 7	6.15 (3.08) N = 15	1.91 (1.85) *N* = 5
**Standardized hair concentration (N = 77)**	0.19 (1.56) *N* = 53^b^	−2.48 (2.28) *N* = 23^b^	0.11 (1.13) *N* = 57	−1.52 (1.39) N = 20

^a^All summaries are reported as means (standard deviations)

^b^One participant was missing viral load information at baseline

Virologic suppression: HIV RNA < 400 copies/mL; virologic failure: HIV RNA ≥400 copies/mL

At baseline, 65% of young people in both study arms were virologically suppressed (Table 2). At six-month follow-up, virologic suppression rate increased to 75% in the intervention arm, with the rate in the SOC arm remaining unchanged (adjusted risk ratio = 1.13; 95% CI = 0.94, 1.36). Comparing intervention to SOC baseline to six-months, a larger reduction in log viral load (i.e., ~ 57% larger reduction (95% CI = − 1.69, 0.01) was noted in the intervention arm (Table 3). Four participants changed from a first-line (NNRTI-based) to second-line (PI-based) regimen from the baseline to six-month visit. Of these, one became suppressed and was in the intervention arm.

## Discussion

This is the first study to demonstrate that an acceptable, feasible, and scalable group-based mental health intervention has the potential to improve adherence and virologic outcomes based on objective measures among a broad age of YPLWH. Few other studies have published evidence for interventions that address mental health challenges to improve HIV outcomes in this population. Studies from the United States [14, 46] including CHAMP+ [47], a US-model using a family intervention focused on pre-adolescents (10–14 years of age) has been adapted for use in South Africa [48] and Thailand [49] and showed promising pilot data for this younger age group. Community adolescent treatment supporters in the CATS intervention used a CATS mentor who trained and supervised older peers (ages 18–24 years) that paired with young adolescents (age 10–14 years) and provided weekly home visits with adherence and psychosocial support [50]. Findings from this CATS intervention indicated improved psychological well-being (confidence, self-esteem, self-worth) and increased self-reported adherence, but the CATS team had no particular scripted content for quality assurance or objective measures of adherence or HIV RNA described in the intervention [50]. The Friendship Bench is a mental health intervention from Zimbabwe that is a promising model using lay counselors to improve mental health, but this intervention did not focus on youth nor HIV outcomes [51]. An intervention targeting older youth living with HIV (age 15–19 years) in southern Africa is planned, but preliminary data is unavailable and enrollment has yet to begin [52]. SYV is unique in its design being specific for the mental health needs of YPLWH in an African context and the use of objective measures of adherence. The intervention was feasible based on excellent attendance and fidelity to the scripted manual. The scripted manual for SYV with training and supervision of lay counselors allows for eventual scalability and reproducibility in settings with few mental healthcare professionals.

The results evaluating trends of effectiveness outcomes in preparation for a larger, fully powered trial, suggest that *SYV* has the potential to improve biologic HIV outcomes. This study enrolled YPLWH regardless of their score on mental health measures or virologic status, assuming all young people could benefit from the intervention by gaining life skills and resilience [22]. The broad inclusion criteria may have limited our ability to demonstrate significant improvement on the current measures, and a larger trial may further target those with virologic non-suppression and mental health challenges. Finally, those randomized to the SOC arm demonstrated higher PHQ-9 scores at baseline, but levels at six-months were comparable between arms. Reasons for the improvement in mental health in the SOC arm are unclear. Simply hearing about intervention content from friends in the intervention arm (contamination risk) is unlikely to result in dramatic improvement in mental health in the SOC arm because the delivery and practice of concepts are thought to be critical to intervention success. Simply asking survey questions related to mental health during the study visits may have a positive impact. Regression to the mean whereby the SOC arm had more room for improvement over time may also explain the reduction in mental health symptoms.

**Fig. 2:**
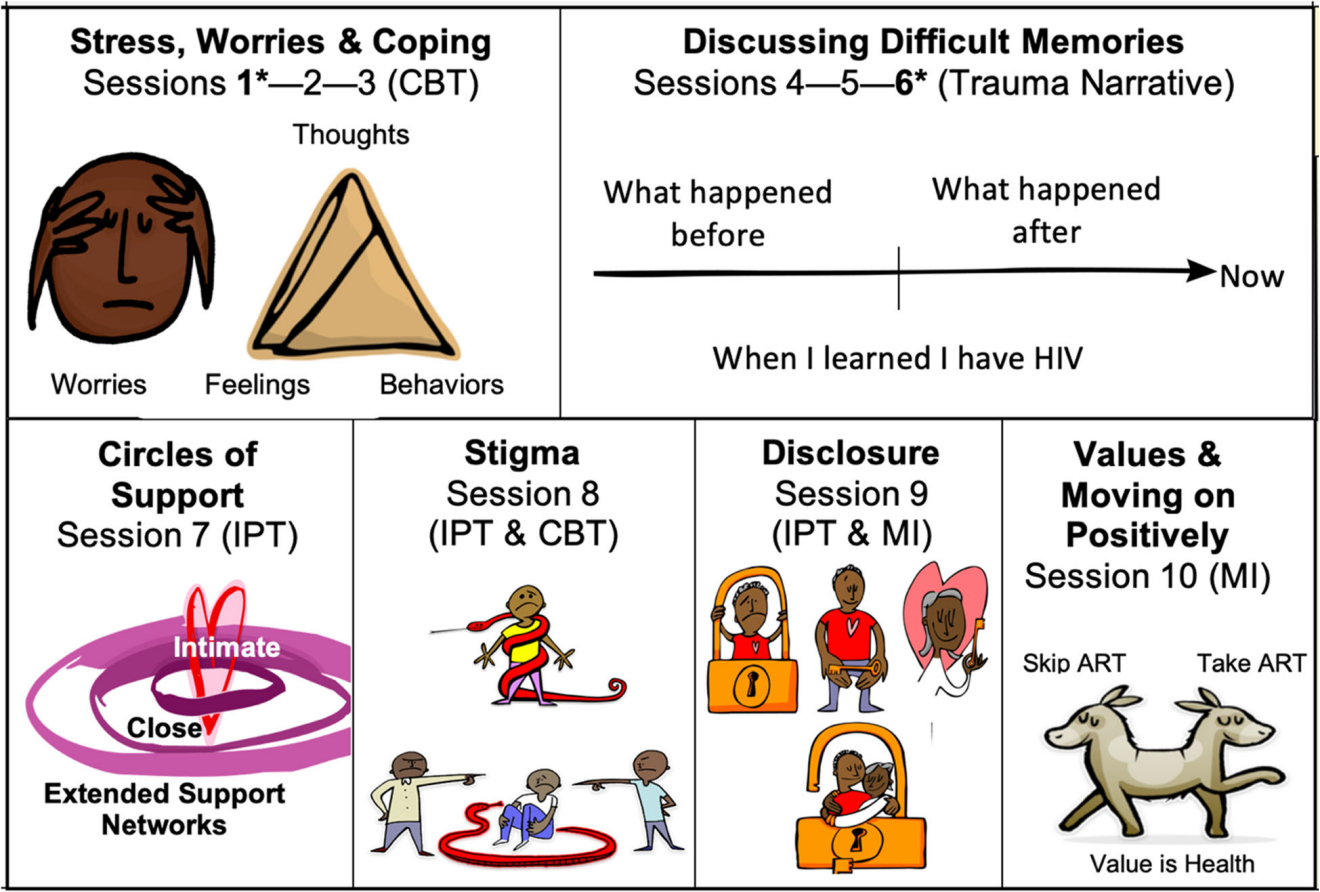
Sauti ya Vijana (The Voice of Youth) Group Session Content. CBT: Cognitive Behavioral Therapy; IPT: Interpersonal psychotherapy; MI: motivational interviewing. *held jointly with caregivers. Images created by SYV illustrator, Imraan Osman, using Adobe Creative Suite 5 Photoshop, 2010

HIV stigma was pervasive among YPLWH in both study arms. Based on our formative work, one full group session was devoted entirely to internal and external stigma, though the topic arose naturally in every session. In session 8, stigma was represented as a snake (Fig. 2), and the CBT Triangle was used to help youth challenge their negative thoughts regarding internal stigma and to consider why people in the community may stigmatize others. Participants discussed how to educate others in order to reduce stigma, but the youth talked about how they feared actually educating others about HIV for fear of their own HIV status being disclosed. Both study arms reported reduced internal stigma at six-months; however, the intervention group reported increased external stigma, while the SOC arm reported reduced. The SYV arm may have experienced heightened awareness of external stigma due to ongoing discussions or, alternatively, the intervention may have inadvertently exacerbated external stigma. Youth reported feeling more comfortable with their HIV status based on the qualitative reports in their final individual session (reduced internal stigma), but ongoing education will be important to reduce stigma in their community.

Self-reported ART adherence improved significantly in the intervention arm; however, this could be due to social desirability bias given weekly discussions of the importance of ART adherence. To quantify adherence objectively, ART concentrations in hair samples were used as a cumulative adherence measure over time. The intervention arm did show trends of both increased ART hair levels as well as reduced log viral loads. Indeed, 10% more participants in the intervention arm were virologically suppressed at six-months compared to SOC participants; however, these differences were not statistically significant. When interpreting ART concentration and virologic outcome, it is important to consider that fluctuating levels above or below a “therapeutic threshold” may not result in change in virologic outcome (Supplemental Figure 2) and HIV resistance mutations may result in virologic failure despite adequate adherence [53, 54].

The study had several limitations. Those randomized to the SOC arm did not meet in groups like the intervention arm, and attendance to the six-month study visit was worse in the SOC arm compared to the SYV arm. The mental health screening tools were not specifically validated in this population or in this cultural context. This can make interpretation of cut-off scores challenging. For that reason, we included both the cutoff and continuous measures.

Based on study findings, the mechanism of change by which the intervention may be effective could be mediated by improved resilience or coping that were not directly measured in this pilot trial but were described qualitatively by many participants during their final individual session. Questionnaires were conducted via a structured interview with a research assistant, possibly introducing social desirability bias and may be better conducted on a computer or tablet device. Challenges to obtaining and interpreting the biologic measure of ART adherence included participants having very short or shaved hair making the sample inadequate for testing, research assistants failing to mark the distal end of longer hair whereby the proximal end (closest to the hair root) has the highest concentration, and ART regimen switches limiting time with drug exposure due to pharmacy stock outs or provider decisions that were not well documented in the clinical file, all leading to a smaller denominator (*N* = 77) and further limiting power for this outcome. Enrollment more than six -months before the start of an intervention wave was problematic due to time lag in that some young people were no longer available to participate on weekly Saturdays. Use of computer-generated block randomization on the day of intervention may improve balance of the intervention and SOC arms in a future effectiveness trial. The study intervention does not address issues of poverty that impact YPLWH, 25% of whom were both out of school and unemployed. Incorporating sustainable income-generating activities may be a useful component for future intervention work. Finally, young people were enrolled from the adolescent HIV clinic, missing the opportunity to reach YPLWH not engaged in care. Nonetheless, the feasibility, acceptability, scalability and trends towards effectiveness of SYV provide compelling evidence to support testing the scalability and effectiveness of this intervention in a fully powered trial.

## Conclusions

The number of YPLWH in Africa, where mental health resources are scarce, continues to rise. An evidence-based mental health intervention is urgently needed to address the unique mental health challenges and poor HIV outcomes in YPLWH. The SYV intervention offers promise to improve mental health, adherence, and virologic suppression in this important and vulnerable population of YPLWH.

## Supplementary information

**Additional file 1: Supplemental Table 1.** Participant Demographics at Study Enrollment. Table shows the demographic differences between those 128 enrolled, 105 randomized, and 93 included in the manuscript

**Additional file 2: Supplemental Table 2.** Fidelity checklist for youth group introduction to the program. Both reviewers asked details about fidelity measurement and this is included as an example.

**Additional file 3: Supplemental Figure 1.** Sauti ya Vijana, Stepped-Wedge Study Design with Average and Age Range Represented in Each Group. ^+^Mean age and in parenthesis (age range) in years rounded to the whole number. The SOC mean age and range is reflected in the SYV intervention group. Abbreviations: SYV = Sauti ya Vijana intervention; SOC = standard of care “control” group; mo = month. Baseline includes 88 participants; an additional 40 participants enrolled over time to complete groups for waves 2 and 3. Shading: Wave 1 in pink, Wave 2 in orange (note, no male SOC group), Wave 3 in green with study follow visits shaded for those who received intervention and remaining clear for the control group; partial shading signifies one or more participants, but not all crossed over. ^*^First crossover wave in blue (3 males from W1; 4 males from W3; 2 females W2 all from KCMC site); ^**^Second crossover wave in purple (run as a mixed gender and mixed site group). Study questionnaire and outcome measures at entry/baseline, 6-month follow-up (+/− 6 weeks due to school exams and holidays) are in corresponding color blocks to indicate data in the present manuscript; 12-, 18- and 30-mo follow up, and cross over data are currently being analyzed.

**Additional file 4: Supplemental Figure 2.** Spaghetti plots of each antiretroviral therapy concentration by virologic outcome. A green dot represents HIV RNA < 400 copies/mL. A red dot represents HIV RNA ≥ 400 copies/mL. The line joining the baseline to 6-month measure matches the color of the HIV RNA outcome at 6-months. Panel A: nevirapine; Panel B: efavirenz; Panel C: lopinavir; Panel D: atazanavir.

## Data Availability

The datasets generated during and analyzed during the current study are available from the corresponding author on request.

## Abbreviations

AIDS: acquired immunodeficiency syndrome
ART: antiretroviral therapy
ATV: atazanavir
BLQ: below limit of quantitation
CATS: community adolescent treatment supporters
CI: confidence interval
EFV: efavirenz
HIV: human immunodeficiency virus
KCMC: Kilimanjaro Christian Medical Centre
LC-MS/MS: liquid chromatography–tandem mass spectrometry
LPV: lopinavir
MRRH: Mawenzi Regional Referral Hospital
NNRTI: Non-nucleoside reverse-transcriptase inhibitors
NVP: nevirapine
PHQ-9: Patient Health Questionnaire
PI-base: protease inhibitor
RTV: ritonavir
SD: standard deviations
SDQ: Strengths and Difficulties Questionnaire
SOC: standard of care
SYV: Sauti ya Vijana
UCSF HAL: the University of California, San Francisco Hair Analytical Laboratory
YPLWH: young people living with HIV
