# Supplementary material for: A group-based mental health intervention for young people living with HIV in Tanzania: results of a pilot individually randomized group treatment trial
Source: BMC Public Health. 2020 Sep 4;20:1358. doi: 10.1186/s12889-020-09380-3 (PMC7487650; doi:10.1186/s12889-020-09380-3)
Supplement: Supplementary file 1 — Additional file 1: Supplemental Table 1. Participant Demographics at Study Enrollment. Table shows the demographic differences between those 128 enrolled, 105 randomized, and 93 included in the manuscript [file 12889_2020_9380_MOESM1_ESM.docx]

**Supplemental Table 1: Participant Demographics at Study Enrollment**

|  | | Enrolled  N=128 | Randomized  N=105 | Participants with 6-month follow-up  N=93 |
| --- | --- | --- | --- | --- |
| Gender (female) | | 68 (53%) | 56 (53%) | 47 (51%) |
| Age* | | 17.9 (2.8) | 18.1 (2.3) | 18.1 (2.3) |
| Primary  Caregiver | Biological parent | 61 (48%) | 51 (49%) | 47 (51%) |
|  | Grandparent | 20 (16%) | 15 (14%) | 14 (15%) |
|  | Aunt/Uncle | 35 (27%) | 28 (27%) | 23 (25%) |
|  | Sibling | 6 (5%) | 5 (5%) | 5 (5%) |
|  | Other | 6 (5%) | 6 (6%) | 4 (4%) |
| Orphan status | Both parents alive | 28 (22%) | 26 (25%) | 23 (25%) |
|  | Single orphan | 64 (53%) | 55 (52%) | 50 (56%) |
|  | Double orphan | 32 (25%) | 24 (23%) | 20 (22%) |
| Perinatal HIV Transmission | | 112 (88%) | 90 (86%) | 78 (84%) |
| Livelihood | In School | 85 (66%) | 68 (65%) | 65 (70%) |
|  | Working | 10 (8%) | 9 (9%) | 11 (12%) |
|  | Both | 4 (3%) | 3 (3%) | 3 (3%) |
|  | Neither | 29 (23%) | 25 (24%) | 23 (25%) |
| Home  Environment | Electricity only | 22 (17%) | 17 (16%) | 14 (15%) |
|  | Indoor plumbing only | 12 (9%) | 9 (9%) | 7 (8%) |
|  | Both | 71 (56%) | 61 (58%) | 54 (58%) |
|  | Neither | 23 (18%) | 18 (17%) | 18 (19%) |
| Own a Cell Phone | | 83 (65%) | 69 (66%) | 62 (67%) |
| Behaviors | Report sexual activity | 42 (33%) | 30 (29%) | 27 (29%) |
|  | Age at sexual debut* | 16.5 (3.3) | 16.6 (3.3) | 16.4 (3.3) |
|  | Reports condom use with latest sexual encounter** | 28 (67%) | 21 (70%) | 19 (70%) |
|  | Report consuming alcohol/other drugs | 13 (10%) | 11 (11%) | 8 (9%) |
| Mental Health | PHQ9 | 5.8 (4.2) | 5.8 (3.9) | 5.5 (3.7) |
|  | Cutoff > 10 | 21 (17%) | 16 (15%) | 14 (15%) |
|  | SDQ | 7.3 (3.8) | 7.3 (3.8) | 7.2 (3.8) |
|  | Cutoff > 17 | 1 (1%) | 0 (0%) | 0 (0%) |
|  | Trauma | 9.4 (7.4) | 9.1 (7.4) | 8.9 (7.2) |
|  | Cutoff > 18 | 22 (17%) | 17 (16%) | \| 14 (15%) \| \| --- \| |
| Stigma | Total | 22.5 (4.8) | 22.6 (4.8) | 22.6 (4.6) |
|  | Internal | 8.0 (2.0) | 8.0 (1.9) | \| 7.9 (1.9) \| \| --- \| |
|  | External | 14.5 (4.2) | 14.7 (4.1) | 14.9 (4.1) |
| Self-report adherence | | 58.2 (13.2) | 59.3 (13.3) | 59.4 (13.2) |
| HIV RNA log_10_ (copies/mL) | | 5.1 (3.0) | 5.2 (3.1) | 5.3 (3.1) |
| HIV RNA (<400 copies/mL) | | 84 (68%) | 70 (67%) | 60 (65%) |

*mean (SD) is standard deviation ; **denominator is those who report sexual activity
