# Supplementary material for: A group-based mental health intervention for young people living with HIV in Tanzania: results of a pilot individually randomized group treatment trial
Source: BMC Public Health. 2020 Sep 4;20:1358. doi: 10.1186/s12889-020-09380-3 (PMC7487650; doi:10.1186/s12889-020-09380-3)
Supplement: Supplementary file 2 — Additional file 2: Supplemental Table 2. Fidelity checklist for youth group introduction to the program. Both reviewers asked details about fidelity measurement and this is included as an example. [file 12889_2020_9380_MOESM2_ESM.docx]

| Group activity  Supplemental Table 3: Fidelity checklist for youth group Introduction to the program | Check if done | Time estimate in minutes/  Time actually used | Comments for supervision, if needed. Note activities that may need repeating or carried forward to next session. |
| --- | --- | --- | --- |
| Start time |  | | |
| **Youth and caregivers jointly** | | | |
| Greeting: Greet each youth and caregiver by name and check attendance. |  | 15/ |  |
| Getting to know each other: Group activity |  | 10/ |  |
| Introduction to the program: Discuss and distribute YG Handout 1. |  | 20/ |  |
| Discuss attendance, travel reimbursement and consequences for being late. |  |  |  |
| Emphasize the importance of confidentiality and sign the confidentiality agreement |  |  |  |
| **Youth only** | | | |
| New topic: Introduce common stresses and worries that can come living with HIV/AIDS. |  | 25/ |  |
| Summarize what other youth report about some of the difficulties and worries they face living with HIV/AIDS (Use YG Handout 2). |  |  |  |
| Use group activity for identifying stresses and  Worries |  |  |  |
| Normalize and validate stresses and worries. |  |  |  |
| Instill hope that the program can work. |  |  |  |
| Homework assigned: Emphasize that home work is important and why. |  | 10/ |  |
| Fun time: After reunited with caregivers |  | 10/ |  |
| **Caregivers only** | | | |
| New topic: Discuss with caregivers what the youth groups will do. |  | 35/ |  |
| Explain that the group leaders will work with the youth about living positively and taking care of their health so they can live long and well. |  |  |  |
| Discuss what group leaders and youth need from the caregivers. |  |  |  |
| Support the caregiver and their own needs. |  |  |  |
| More time can be used for caregivers to discuss among themselves. |  | Open |  |
| Fun time: Activity with youth, caregivers, and group leaders together |  | 10/ | |
| End time |  | | |
