# Supplementary material for: A group-based mental health intervention for young people living with HIV in Tanzania: results of a pilot individually randomized group treatment trial
Source: BMC Public Health. 2020 Sep 4;20:1358. doi: 10.1186/s12889-020-09380-3 (PMC7487650; doi:10.1186/s12889-020-09380-3)
Supplement: Supplementary file 3 — Additional file 3: Supplemental Figure 1. Sauti ya Vijana, Stepped-Wedge Study Design with Average and Age Range Represented in Each Group. +Mean age and in parenthesis (age range) in years rounded to the whole number. The SOC mean age and range is reflected in the SYV intervention group. Abbreviations: SYV = Sauti ya Vijana intervention; SOC = standard of care “control” group; mo = month. Baseline includes 88 participants; an additional 40 participants enrolled over time to complete groups for waves 2 and 3. Shading: Wave 1 in pink, Wave 2 in orange (note, no male SOC group), Wave 3 in green with study follow visits shaded for those who received intervention and remaining clear for the control group; partial shading signifies one or more participants, but not all crossed over. *First crossover wave in blue (3 males from W1; 4 males from W3; 2 females W2 all from KCMC site); **Second crossover wave in purple (run as a mixed gender and mixed site group). Study questionnaire and outcome measures at entry/baseline, 6-month follow-up (+/− 6 weeks due to school exams and holidays) are in corresponding color blocks to indicate data in the present manuscript; 12-, 18- and 30-mo follow up, and cross over data are currently being analyzed. [file 12889_2020_9380_MOESM3_ESM.docx]

| Months | | July-Aug | Sep - Dec | Jan | Feb-May | Jun-July | Aug-Nov | Dec-Jan | | Feb-Apr | May-July | Dec-Jan | | May-Aug | | Dec-Jan | |
| --- | --- | --- | --- | --- | --- | --- | --- | --- | --- | --- | --- | --- | --- | --- | --- | --- | --- |
| Year | | 2016 | | 2017 | | | | | 2018 | | | | 2019 | | | | 2020 |
| Wave 1 |  | **Entry/ Baseline** |  | **6-mo** |  | **12-mo** |  | **18-mo** | |  |  | **30-mo** | |  | |  | |
|  | Male SYV | x | SYV Int | x |  | x |  | x | |  |  | x | |  | |  | |
|  | Female SYV | x |  | x |  | x |  | x | |  |  | x | |  | |  | |
|  | Male SOC | x |  | x |  | x |  | x | | SYV Int^^ | x | x | | x | SYV Int^γ^ |  | |
|  | Female SOC | x |  | x |  | x |  | x | |  |  | x | | x |  |  | |
| Wave 2 |  | **Entry** |  | **Baseline** |  | **6-mo** |  | **12-mo** | |  | **18-mo** |  | | **30-mo** | |  | |
|  | Male SYV | x |  | x | SYV Int | x |  | x | |  | x |  | | x | |  | |
|  | Female SYV | x |  | x |  | x |  | x | |  | x |  | | x | |  | |
|  | Female SOC | x |  | x |  | x |  | x | | SYV Int^^ | x | x | | x | SYV Int^γ^ |  | |
| Wave 3 |  | **Entry** |  |  |  | **Baseline** |  | **6-mo** | |  | **12-mo** | **18-mo** | |  | | **30-mo** | |
|  | Male SYV | x |  |  |  | x | SYV Int | x | |  | x | x | |  | | x | |
|  | Female SYV | x |  |  |  | x |  | x | |  | x | x | |  | | x | |
|  | Male SOC | x |  |  |  | x |  | x | | SYV Int^^ | x | x | | x | SYV Int^γ^ | x | |
|  | Female SOC | x |  |  |  | x |  | x | |  | x | x | | x |  | x | |

**Supplemental Figure 1: Sauti ya Vijana, Stepped-Wedge Study Design**

Abbreviations: SYV= Sauti ya Vijana intervention; SOC= standard of care “control” group; mo=month. Baseline includes 88 participants; an additional 40 participants enrolled over time to complete groups for waves 2 and 3.

Shading: Wave 1 in pink, Wave 2 in orange (note, no male SOC group), Wave 3 in green with study follow visits shaded for those who received intervention and remaining clear for the control group; partial shading signifies one or more participants, but not all crossed over. ^^First crossover wave in blue (3 males from W1; 4 males from W3; 2 females W2 all from KCMC site); ^γ^Second crossover wave in purple (run as a mixed gender and mixed site group)

^*^Study questionnaire and outcome measures at baseline, pre, 6-month follow-up (+/- 6 weeks due to school exams and holidays) are in corresponding color blocks to indicate data in the present manuscript; 12-, 18- and 30-mo follow up, and cross over data currently being analyzed.
