# Supplementary material for: A group-based mental health intervention for young people living with HIV in Tanzania: results of a pilot individually randomized group treatment trial
Source: BMC Public Health. 2020 Sep 4;20:1358. doi: 10.1186/s12889-020-09380-3 (PMC7487650; doi:10.1186/s12889-020-09380-3)
Supplement: Supplementary file 4 — Additional file 4: Supplemental Figure 2. Spaghetti plots of each antiretroviral therapy concentration by virologic outcome. A green dot represents HIV RNA < 400 copies/mL. A red dot represents HIV RNA ≥ 400 copies/mL. The line joining the baseline to 6-month measure matches the color of the HIV RNA outcome at 6-months. Panel A: nevirapine; Panel B: efavirenz; Panel C: lopinavir; Panel D: atazanavir. [file 12889_2020_9380_MOESM4_ESM.docx]

| 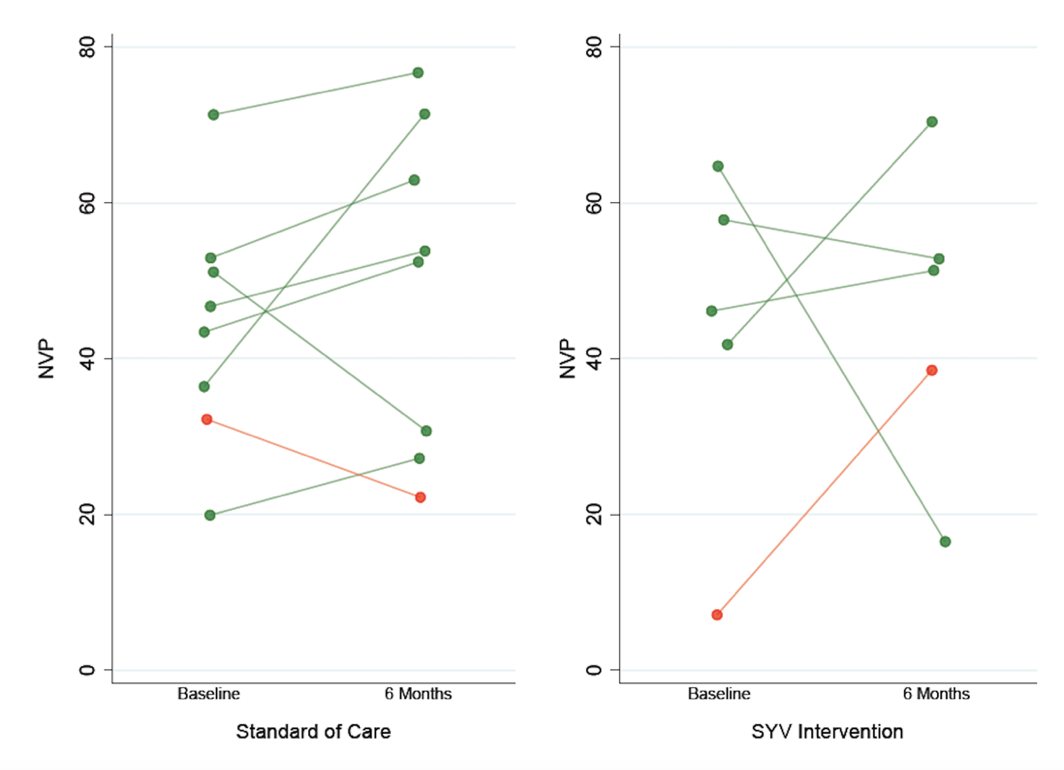A | 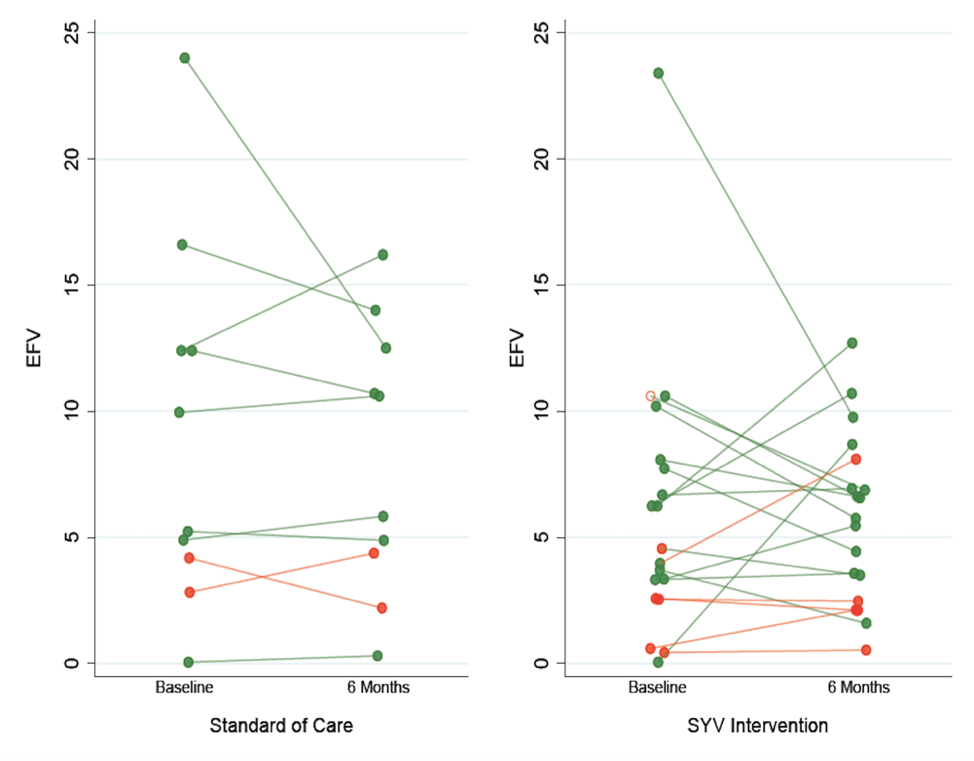B |
| --- | --- |
| 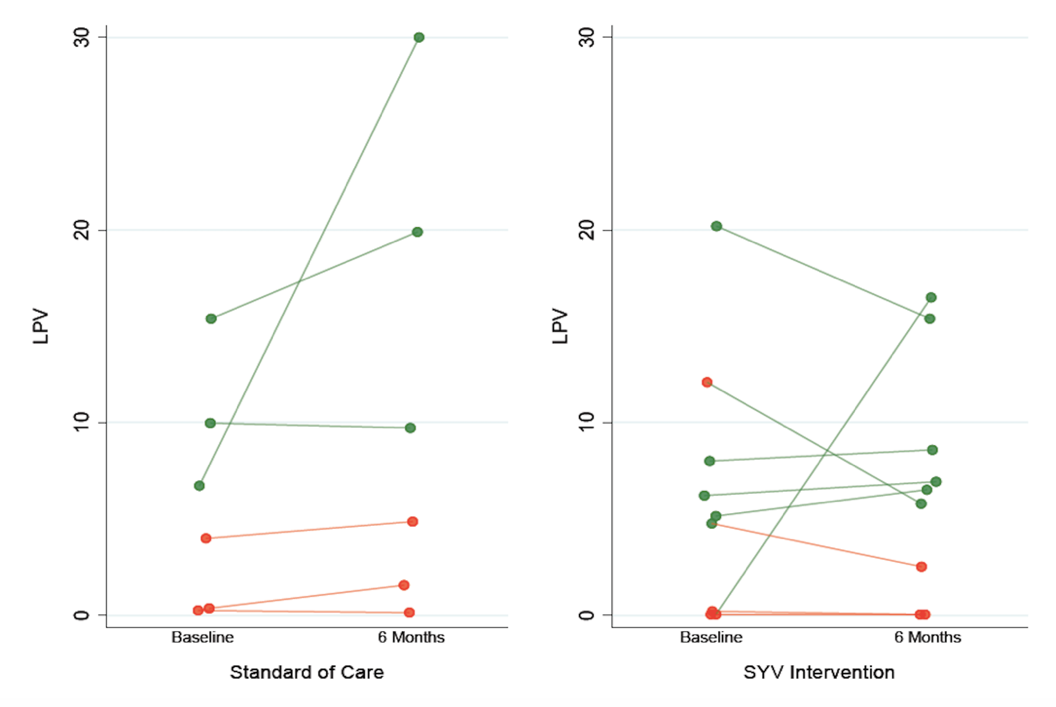C | 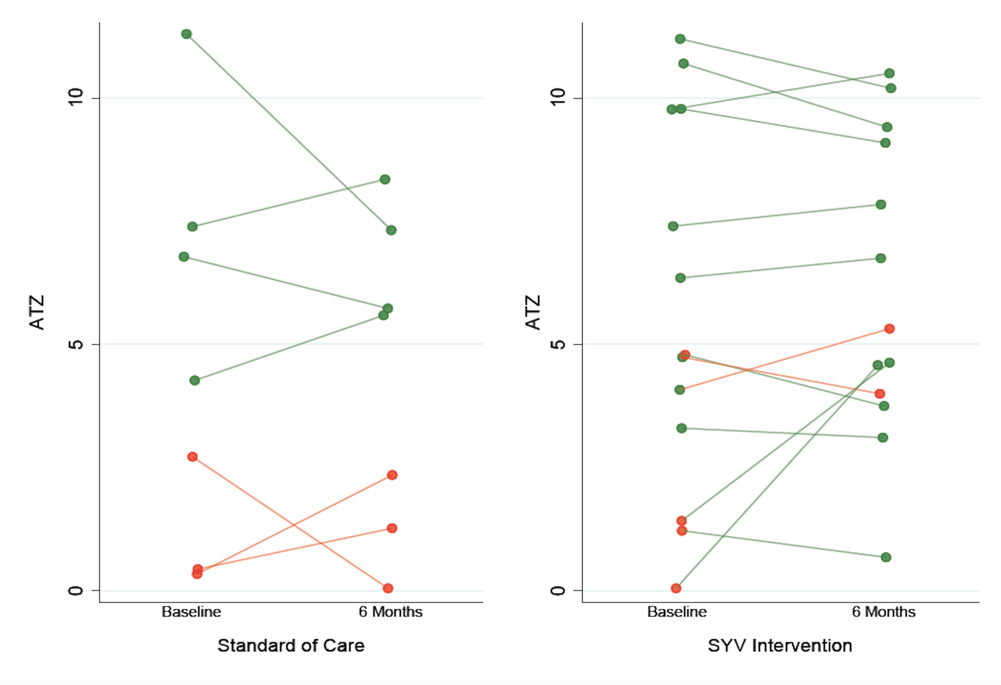  D |

**Supplemental Figure 2: Spaghetti plots of each antiretroviral therapy concentration by virologic outcome.**

A green dot represents HIV RNA <400 copies/mL. A red dot represents HIV RNA > 400 copies/mL. The line joining the baseline to 6-month measure matches the color of the HIV RNA outcome at 6-months. Panel A: nevirapine; Panel B: efavirenz; Panel C: lopinavir; Panel D: atazanavir.
